# Videourodynamic Studies of Women with Voiding Dysfunction

**DOI:** 10.1038/s41598-017-07163-2

**Published:** 2017-07-28

**Authors:** Sheng-Mou Hsiao, Ho-Hsiung Lin, Hann-Chorng Kuo

**Affiliations:** 1 Department of Obstetrics and Gynecology, Far Eastern Memorial Hospital, Banqiao, New Taipei Taiwan; 2 Graduate School of Biotechnology and Bioengineering, Yuan Ze University, Taoyuan, Taiwan; 3 Department of Obstetrics and Gynecology, National Taiwan University Hospital, Taipei, Taiwan; 4 Department of Urology, Buddhist Tzu Chi General Hospital and Tzu Chi University, Hualien, Taiwan

## Abstract

This retrospective study is aimed to present videourodynamic findings of women with symptoms of voiding dysfunction in a medical center. Of 1914 women, the diagnoses included bladder outlet obstruction (BOO, n = 810, 42.3%), bladder dysfunction (n = 1,048, 54.8%) and normal tracings (n = 56, 2.9%). Anatomic BOO (n = 49) included cystocele (n = 19) and urethral stricture (n = 30). Common functional BOOs included dysfunctional voiding (n = 325, 17.0%) and poor relaxation of the external sphincter (n = 336, 17.6%). Common bladder dysfunction subtypes included detrusor underactivity (n = 337, 17.6%), detrusor hyperactivity with impaired contractility (n = 231, 12.1%), and bladder oversensitivity (n = 325, 17.0%). Receiver operating characteristic (ROC) analysis were performed, and the following optimum cutoff values were determined: (1) voiding detrusor pressure at a maximum flow rate (Pdet.Qmax) = 30 cmH_2_O for differentiating BOO from bladder dysfunction and normal tracings, with an ROC area of 0.78; (2) the Abrams-Griffiths number = 30 for differentiating anatomic from functional BOO, with an ROC area of 0.66; (3) post-void residual = 200 mL for differentiating bladder neck dysfunction from the other BOOs, with an ROC area of 0.69; (4) Pdet.Qmax = 30 cmH_2_O for differentiating dysfunctional voiding from poor relaxation of the external sphincter with an ROC area of 0.93. **T**he above findings can be used as initial guide for management of female BOO.

## Introduction

Clinical lower urinary tract symptoms of women are not reliable for the diagnosis of lower urinary tract dysfunction^1^. Symptoms-based treatment may be ineffective and may also expose patients to unnecessary treatment-related adverse effects. Pressure flow urodynamic studies can be used to diagnose bladder outlet obstruction (BOO)^2^. However, the management of BOO may differ among its subgroups^3^. Videourodynamics can provide a definitive diagnosis for women with voiding symptoms^4–7^. Nonetheless, many urodynamic units do not have fluoroscopy, and fluoroscopy would result in a small but significant radiation exposure for the patients, technicians or even physicians.

Thus, it is preferred to make a differential diagnosis in women with voiding symptoms using test without radiation, such as uroflowmetry or urodynamic studies, thereby avoiding radiation exposure. Therefore, the main aim of this study is to determine if any parameters of urodynamic studies can be used as initial guide for the differential diagnosis of women with voiding dysfunction. In addition, the main cause of female BOO is controversial^7, 8^, we also aim to present the videourodynamic findings in women with symptoms of voiding dysfunction.

## Results

**Figure 1:**
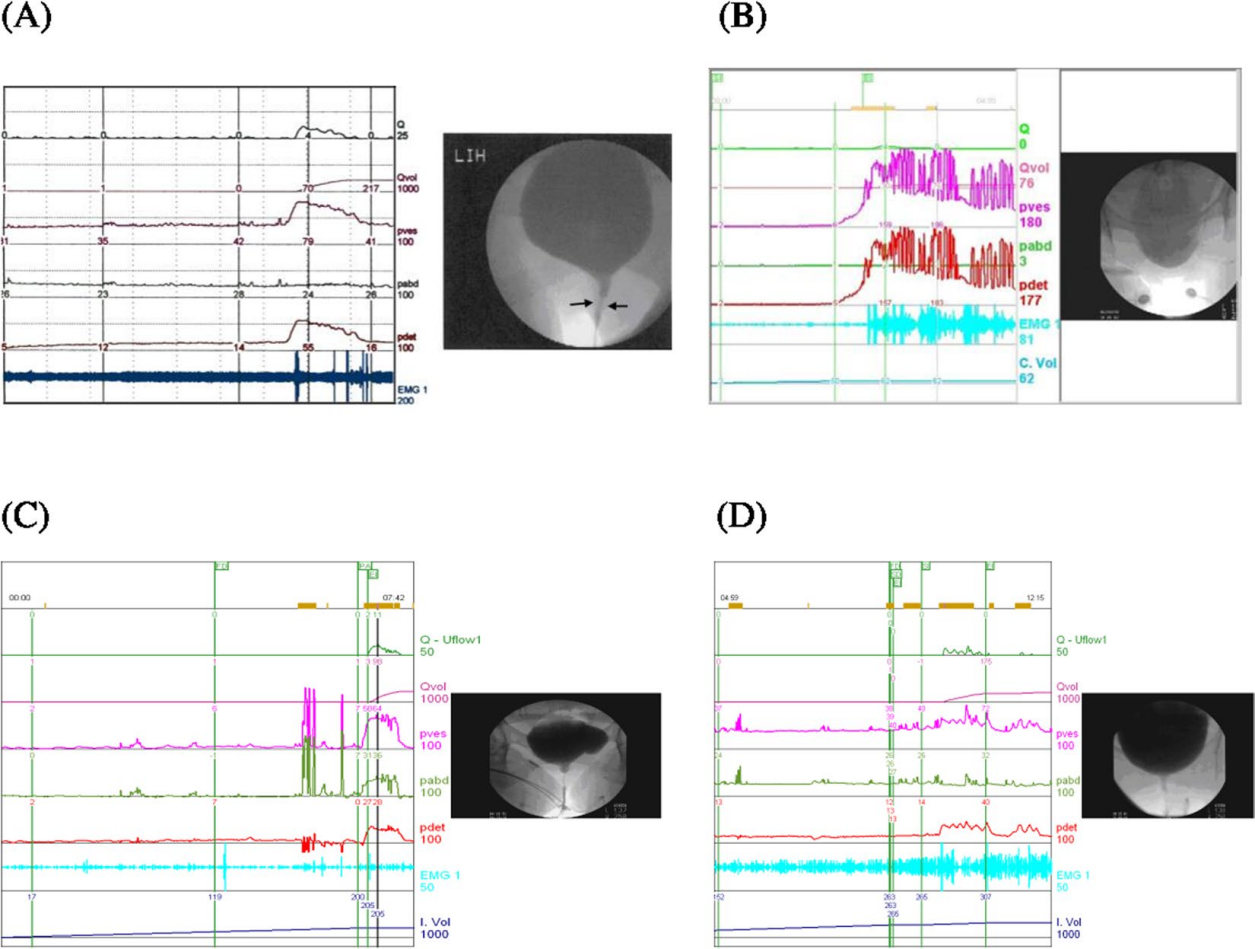
Videourodynamic findings of the subtypes of bladder outlet obstruction: (**A**) urethral stricture, (**B**) bladder neck dysfunction, (**C**) dysfunctional voiding and (**D**) poor relaxation of the external sphincter.

Of 1914 women, the diagnoses included bladder outlet obstruction (BOO, n = 810, 42.3%), bladder dysfunction (n = 1,048, 54.8%) and normal tracings (n = 56, 2.9%) (Table 1). BOO included functional and anatomic BOOs. Anatomic BOO included urethral stricture (n = 30, Fig. 1A) and cystocele (n = 19). Functional BOOs included bladder neck dysfunction (n = 100, 5.2%, Fig. 1B), dysfunctional voiding (n = 325, 17.0%, Fig. 1C) and poor relaxation of the external sphincter (n = 336, 17.6%, Fig. 1D) (Table 2). The most common types of bladder dysfunction were detrusor underactivity (n = 337, 17.6%), bladder oversensitivity (n = 325, 17.0%) and detrusor hyperactivity and impaired contractility (n = 231, 12.1%).

**Table 1:** Comparisons of videourodynamic diagnoses in women with voiding dysfunction symptoms (n = 1,914).

Variables	BOO (n = 810)	Bladder dysfunction (n = 1,048)	Normal tracings (n = 56)	P^a^	BOO vs. Bladder dysfunction, P^b^
Age (years)	59.3 ± 16.7	64.7 ± 16.2	54.0 ± 14.3	0.0001	<0.0001
Detrusor overactivity	282 (35)	306 (29)	0 (0)	0.001	0.01
First sensation of filling (mL)	141 ± 69	157 ± 92	167 ± 72	0.003	0.02
Full sensation (mL)	223 ± 97	235 ± 117	290 ± 103	0.0001	0.26
Bladder compliance (mL/cmH_2_O)	71.7 ± 84.8	63.5 ± 78.9	84.8 ± 72.0	0.001	0.0003
Voided volume (mL)	202 ± 142	152 ± 120	489 ± 114	0.0001	<0.0001
Post-void residual (mL)	126 ± 139	176 ± 189	20 ± 29	0.0001	<0.0001
Bladder capacity (mL)	329 ± 150	328 ± 154	508 ± 120	0.0001	0.31
Pdet.Qmax (cmH_2_O)	33 ± 23	14 ± 11	17 ± 8.2	0.0001	<0.0001
Qmax (mL/s)	8.9 ± 6.2	7.8 ± 6.5	24.1 ± 7.8	0.0001	<0.0001
Abrams-Griffith number	15.6 ± 26.3	−1.6 ± 14.4	−31.0 ± 17.5	0.0001	<0.0001
Voided volume/bladder capacity (%)	0.63 ± 0.32	0.53 ± 0.37	0.96 ± 0.05	0.0001	<0.0001
Hypertension	161(20)	269 (26)	6 (11)	0.04	0.04
Diabetes	162 (20)	166 (16)	8 (14)	0.053	0.02
Coronary artery disease	23 (3)	41 (4)	2 (4)	0.45	0.21
Chronic kidney disease	19 (2)	21 (2)	2 (4)	0.68	0.62
Chronic obstructive pulmonary disease	13 (2)	9 (1)	0 (0)	0.23	0.14
Moderate/severe voiding symptoms	501 (62)	624 (60)	18 (32)	<0.001	0.31
Urgency/UUI	497 (61)	550 (52)	39 (70)	<0.001	<0.001
Frequency/nocturia	733 (90)	856 (82)	49 (88)	<0.001	<0.001
Urinary retention	88 (11)	233 (22)	3 (5)	<0.001	<0.001

Data were expressed as the mean ± standard deviation or number (percentage). BOO = bladder outlet obstruction; Pdet.Qmax = voiding detrusor pressure at Qmax; Qmax = maximum flow rate; and UUI = urgency urinary incontinence.

^a^By Kruskal-Wallis test or chi-square test.

^b^By Wilcoxon rank-sum test or chi-square test.

**Table 2:** Comparisons of videourodynamic diagnoses with bladder outlet obstruction (n = 810).

Variables	BOO		P^a^	Functional BOO			P^b^	Dysfunctional voiding vs. PRES, P^a^
	Functional (n = 761)	Anatomic (n = 49)		Bladder neck dysfunction (n = 100)	Dysfunctional voiding (n = 325)	PRES (n = 336)		
Age (years)	59.4 ± 13.8	57.8 ± 16.7	0.81	64.1 ± 17.1	61.1 ± 16.5	56.5 ± 15.9	0.0001	0.0001
Detrusor overactivity	260 (34)	22 (45)	0.13	46 (46)	195 (60)	19 (6)	<0.001	<0.001
First sensation of filling (mL)	141 ± 54	141 ± 57	0.70	138 ± 78	130 ± 69	154 ± 64	0.0001	<0.0001
Full sensation (mL)	223 ± 77	214 ± 80	0.48	209 ± 103	197 ± 95	253 ± 90	0.0001	<0.0001
Bladder compliance (mL/cmH_2_O)	73 ± 55	59 ± 46	0.10	61 ± 70	63 ± 77	85 ± 95	0.0001	<0.0001
Voided volume (mL)	205 ± 116	166 ± 120	0.044	142 ± 143	179 ± 125	248 ± 143	0.0001	<0.0001
Post-void residual (mL)	126 ± 109	131 ± 109	0.58	216 ± 187	121 ± 123	104 ± 126	0.0001	0.03
Bladder capacity (mL)	331 ± 119	297 ± 109	0.18	358 ± 176	300 ± 145	352 ± 143	0.0001	<0.0001
Pdet.Qmax (cmH_2_O)	32 ± 17	49 ± 29	0.006	39 ± 24	46 ± 18	17 ± 12	0.0001	<0.0001
Qmax (mL/s)	9.0 ± 4.7	7.0 ± 4.6	0.005	6.0 ± 5.6	9.4 ± 6.1	9.6 ± 6.2	0.0001	0.23
Abrams-Griffith number	14 ± 19	35 ± 30	0.0002	27 ± 26	27 ± 23	−2 ± 15	0.0001	<0.0001
Voided volume/bladder capacity (%)	0.63 ± 0.28	0.54 ± 0.30	0.06	0.41 ± 0.37	0.62 ± 0.31	0.71 ± 0.30	0.0001	0.0002
Moderate/severe voiding symptoms	463 (61)	38 (78)	0.02	68 (68)	176 (54)	219 (65)	0.004	0.004
Urgency/UUI	472 (62)	25 (51)	0.13	56 (56)	235 (72)	181 (54)	<0.001	<0.001
Frequency/nocturia	694 (91)	39 (80)	0.007	89 (89)	292 (90)	313 (93)	0.23	0.16
Urinary retention	83 (11)	5 (10)	0.88	20 (20)	39 (12)	24 (7)	0.001	0.03

PRES = poor relaxation of the external sphincter. Data expression and other abbreviations are the same as in Table 1.

^a^By Wilcoxon rank-sum test or chi-square test.

^b^By Kruskal-Wallis test or chi-square test.

**Figure 2:**
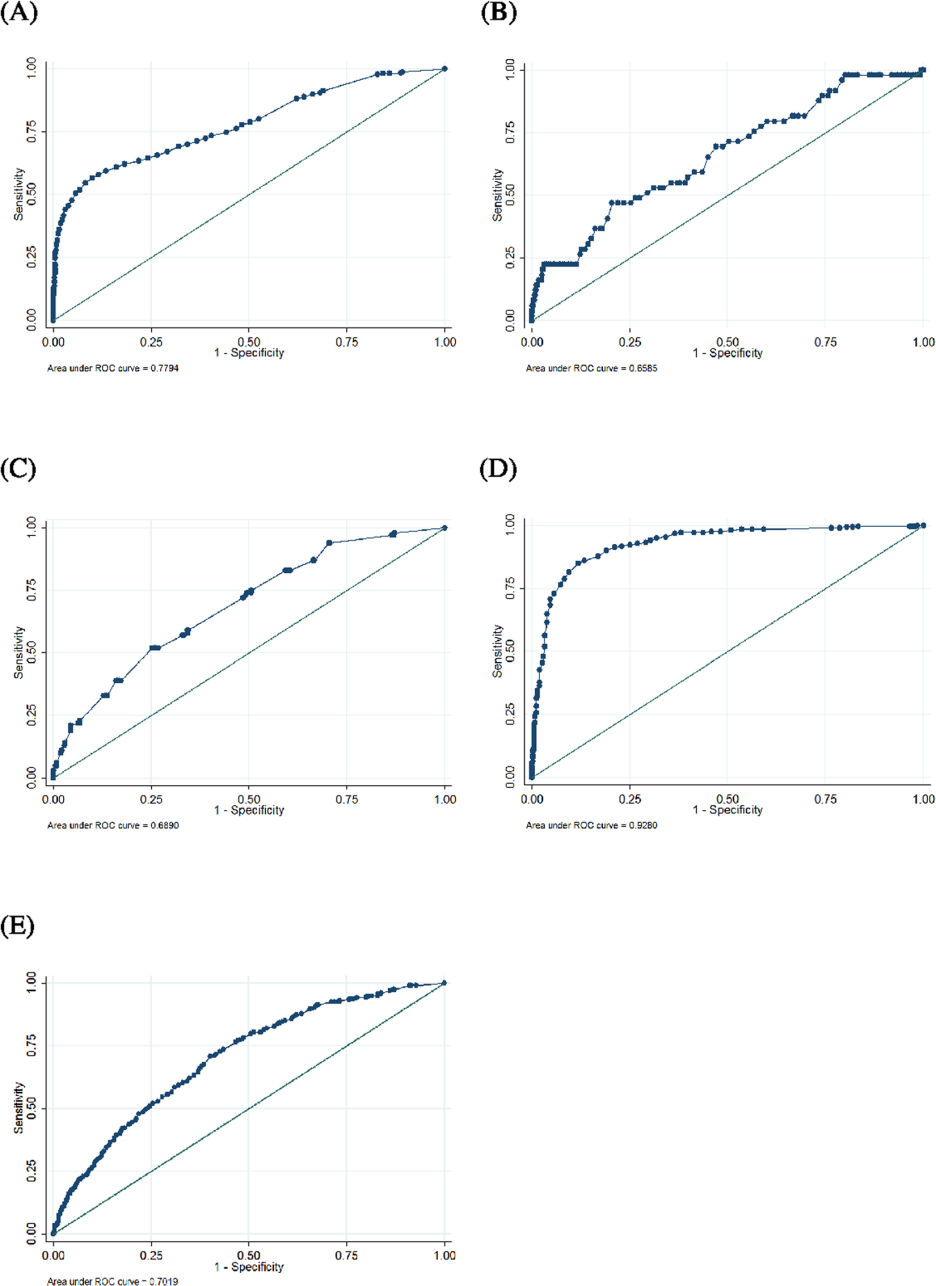
The receiver operating characteristic (ROC) curve of using (**A**) the voiding detrusor pressure at maximum flow rate (Pdet.Qmax) to predict bladder outlet obstruction, (**B**) the Abrams-Griffiths number to predict anatomic bladder outlet obstruction, (**C**) the post-void residual to predict bladder neck dysfunction, (**D**) the Pdet.Qmax to predict dysfunctional voiding, and (**E**) the bladder contractility index to predict bladder dysfunction.

Receiver operating characteristic (ROC) analyses were performed, and the following optimum cutoff values were determined: (1) voiding detrusor pressure at maximum flow rate (Pdet.Qmax)= 30 cmH_2_O for differentiating BOO from bladder dysfunction and normal tracings, with a ROC area of 0.78 (95% confidence interval [CI] = 0.76 to 0.80; sensitivity = 54.6%, specificity = 91.8%) (Fig. 2A); (2) the Abrams-Griffiths number^9^ (i.e., Pdet.Qmax – 2 x maximum flow rate [Qmax]) = 30 for differentiating anatomic BOO from functional BOO, with a ROC area of 0.66 (95% CI = 0.58 to 0.74; sensitivity = 46.9%, specificity = 76.5%) (Fig. 2B); (3) post-void residual = 200 mL, for differentiating bladder neck dysfunction from dysfunctional voiding and poor relaxation of the external sphincter, with a ROC area of 0.69 (95% CI = 0.63 to 0.74; sensitivity = 52.0%, specificity = 74.1%), (Fig. 2C); and (4) Pdet.Qmax = 30 cmH_2_O for differentiating dysfunctional voiding from poor relaxation of the external sphincter, with a ROC area of 0.93 (95% CI = 0.91 to 0.95; sensitivity = 87.7%, specificity = 83.0%) (Fig. 2D).

If we used the bladder contractility index^10^ (i.e., Pdet.Qmax + 5 x Qmax) to predict bladder dysfunction in all women with symptoms of voiding dysfunction, the ROC area was 0.70 (95% CI = 0.68 to 0.73) with a cutoff point of 60 (sensitivity = 70.9%, specificity = 59.7%) (Fig. 2E).

The incidences of moderate/severe voiding symptoms did not differ between the BOO and bladder dysfunction groups (62% and 60%, respectively, P = 0.31, Table 1). Nonetheless, higher incidences of urgency/urgency urinary incontinence and frequency/nocturia, and lower incidence of urinary retention were noted in the BOO group, compared with the bladder dysfunction group (Table 1).

## Discussion

Voiding problems may be due to increased urethral resistance, impaired detrusor contractility or both. We found that only 42.5% of women with symptoms of voiding dysfunction were proven to have anatomic or functional BOO (Table 1). Besides, the incidences of moderate/severe voiding symptoms did not differ between women with BOO and bladder dysfunction (Table 1). Thus, the differential diagnosis for women with symptoms of voiding dysfunction may not depend on clinical symptoms and pelvic examination alone. Urodynamic or videourodynamic studies may be used for differential diagnosis, especially for women who are refractory to medical treatment.

Previously, Groutz *et al*. reported that prior anti-incontinence surgery and severe genital prolapse were the most common etiologies of female BOOs^8^. Brucker *et al*. also reported that anatomic BOO was the main cause of female BOO in their study^7^. However, we found that the most common etiologies for BOO were dysfunctional voiding and poor relaxation of the external sphincter, not anatomic BOO (Table 2).

The cutoff values for uroflowmetry and urodynamic parameters for female BOO are controversial^11–17^. The criterion of Qmax to define female BOO ranges from 11 mL/s to 15 mL/s, and the Pdet.Qmax ranges from 21 cmH_2_O to 50 cmH_2_O^11–16^, and a female BOO nomogram has been reported^17^. Nonetheless, Gravina *et al*. reported that Qmax and Abrams-Griffiths number were the most useful criteria for the diagnosis of urodynamic obstruction, and Pdet.Qmax was the least^18^. However, we found that a Pdet.Qmax ≥ 30 cmH_2_O was the best cutoff value to differentiate BOO from a normal tracing and bladder dysfunction, comparable to our previous report^15^.

The Abrams-Griffiths number has been used to diagnose men with BOO^19^. Using this nomogram, men can be divided into obstructed, equivocal, and unobstructed according to their Abrams-Griffiths number: Abrams-Griffiths number > 40 = obstructed; Abrams-Griffiths number 20–40 = equivocal; and Abrams-Griffiths number < 20 = unobstructed^19^. Gravina *et al*. reported the use of the Abrams-Griffiths number in diagnosing women with BOO^18^. In this study, although the Pdet.Qmax rather than the Abrams-Griffiths number was better to differentiate women with BOO and bladder dysfunction (Fig. 2A), and Abrams-Griffiths number was found to be a potential tool for differential diagnosis of anatomic BOO from functional BOO. However, owing to low ROC area of Abrams-Griffiths number to differentiate anatomic BOO from functional BOO, videourodynamic studies should remain an important tool for differential diagnosis.

Brucker *et al*. reported that Qmax is significantly lower in anatomic BOO^7^. We found that the ROC area of the Abrams-Griffiths number (Fig. 2B) did not differ from the ROC area of Qmax in predicting anatomic BOO (0.66, 95% CI = 0.58 to 0.74 vs. 0.62, 95% CI: 0.54 to 0.70, P = 0.43). Owing to low ROC areas of the Abrams-Griffiths number and Qmax, both parameters should not be used as good tools for differential diagnosis of anatomic and functional BOOs.

The treatment of bladder neck dysfunction may differ from the treatment of dysfunctional voiding and poor relaxation of the external sphincter^3, 20^. Bladder neck dysfunction is caused by a lack of relaxation of the smooth muscles at the bladder neck during the voiding phase. Bladder neck dysfunction, dysfunctional voiding and poor relaxation of the external sphincter can be treated with alpha blockers, clean intermittent catheterization or botulinum A injection^3^; however, their specific treatments may differ. Transurethral incision of the bladder neck can provide definitive treatment for bladder neck dysfunction^3, 20, 21^, while the first-line treatment for dysfunctional voiding and poor relaxation of the external sphincter is pelvic relaxation training^3, 22^. We found that a large post-void residual might be a good parameter in the differential diagnosis of bladder neck dysfunction and dysfunctional voiding/poor relaxation of the external sphincter. We proposed a cutoff value of post-void residual = 200 mL to differentiate bladder neck dysfunction from the dysfunctional voiding/poor relaxation of the external sphincter (Fig. 2C). Thus, for women with urodynamic and clinical findings consistent with BOO, and with a post-void residual > 200 mL, especially for those women refractory to alpha blocker treatment, a videourodynamic study or at minimum a voiding cystourethrogram should be performed to exclude bladder neck dysfunction.

Many authors categorize non-neurologic functional BOO into two types (i.e., bladder neck dysfunction and dysfunctional voiding)^7, 23^, and such dysfunctional voiding can be diagnosed by (1) intermittent and/or fluctuating flow rate due to involuntarily intermittent contractions of the periurethral striated muscle during voiding in a neurologically normal individuals^24^ and (2) urethral dilation to the level of the external sphincter with wide opening of bladder neck^25^; and pelvic floor or striated external sphincter muscle should be responsible for the pathophysiology^24, 26^. We further classified the above diagnosis of dysfunctional voiding into two subtypes (i.e., dysfunctional voiding and poor relaxation of the external sphincter). Although the treatment for women with dysfunctional voiding and poor relaxation of the external sphincter may be similar, we found that the Pdet.Qmax was higher in dysfunctional voiding than in poor relaxation of the external sphincter (mean: 46 vs. 17 cmH_2_O, P < 0.0001) and had a high ROC area (0.93). In addition, the prevalence of detrusor overactivity was low in the poor relaxation of the external sphincter group (6%) compared to the other groups of functional BOO (46% and 60% for bladder neck dysfunction and dysfunctional voiding, respectively). Such a great difference may probably indicate that the chance of finding a diagnosis of poor relaxation of the external sphincter in a BOO woman without detrusor overactivity. Together with the typical videourodynamic findings (i.e., narrowing of the urethra at the middle part in dysfunctional voiding and at the distal part in poor relaxation of the external sphincter, Fig. 1C and D), these findings suggest that dysfunctional voiding and poor relaxation of the external sphincter are different disease entities with different pathogeneses. In brief, the voiding difficulty of dysfunctional voiding is functional BOO; and voiding difficulty of poor relaxation of the external sphincter is the guarding inhibition of the detrusor contraction during voiding attempt.

The bladder contractility index is represented by the following formula: bladder contractility index = Pdet.Qmax + 5 x Qmax^10^. Using this formula, bladder contractility can be divided into strong (>150), normal (100–150), and weak (<100). The mean bladder contractility index was <100 in women with bladder dysfunction. The mean bladder contractility index was highest in women with detrusor overactivity or bladder oversensitivity, intermediate in women with detrusor hyperactivity and impaired contractility, and lowest in women with detrusor underactivity/acontractile detrusor (Table 3). Chancellor reported that the overactive bladder may progress to an underactive bladder^27^. Early education, behavior modification and medical treatment may help to prevent this progression in women with overactive bladder^27^.

**Table 3:** Comparisons of videourodynamic diagnoses with bladder dysfunction (n = 1,048)

Variables	Acontractile detrusor (n = 106)	Detrusor underactivity (n = 337)	DHIC (n = 231)	Detrusor overactivity (n = 49)	Bladder oversensitivity (n = 325)	P^a^
Age (years)	62.5 ± 13.8	67.8 ± 14.5	75.6 ± 9.5	67.5 ± 15.3	54.1 ± 16.0	0.0001
Detrusor overactivity	2 (2)	11 (3)	231 (100)	49 (100)	13 (4)	< 0.001
First sensation of filling (mL)	207 ± 118	196 ± 106	139 ± 72	131 ± 70	118 ± 53	0.0001
Full sensation (mL)	300 ± 132	289 ± 129	196 ± 101	186 ± 92	194 ± 72	0.0001
Bladder compliance (mL/cmH_2_O)	51 ± 87	70 ± 93	58 ± 69	51 ± 55	66 ± 69	0.0001
Voided volume (mL)	81 ± 109	116 ± 123	104 ± 82	230 ± 124	235 ± 86	0.0001
Post-void residual (mL)	331 ± 199	271 ± 198	195 ± 143	28 ± 41	34 ± 75	0.0001
Bladder capacity (mL)	412 ± 162	387 ± 169	299 ± 156	258 ± 125	269 ± 90	0.0001
Pdet.Qmax (cmH_2_O)	4.7 ± 8.1	8.5 ± 9.8	18.0 ± 9.4	20.3 ± 10.1	18.8 ± 9.6	0.0001
Qmax (mL/s)	3.6 ± 5.4	4.9 ± 5.3	6.3 ± 4.4	13.0 ± 8.3	12.4 ± 5.9	0.0001
Abrams-Griffith number	−2.5 ± 12.7	−1.3 ± 12.9	5.4 ± 13.7	−5.7 ± 21.1	−6.0 ± 13.8	0.0001
Voided volume/bladder capacity (%)	0.23 ± 0.30	0.34 ± 0.34	0.36 ± 0.22	0.88 ± 0.14	0.88 ± 0.20	0.0001
Bladder contractility index	22.7 ± 29.1	33.2 ± 30.2	49.6 ± 22.9	85.2 ± 40.9	80.7 ± 32.2	0.0001
Moderate/severe voiding symptoms	90 (85)	273 (81)	143 (62)	17 (35)	101 (31)	<0.001
Urgency/UUI	29 (27)	87 (26)	194 (84)	43 (88)	197 (61)	<0.001
Frequency/nocturia	67 (63)	274 (81)	174 (75)	39 (80)	302 (93)	<0.001
Urinary retention	40 (38)	117 (35)	60 (26)	3 (6)	13 (4)	<0.001

DHIC = detrusor hyperactivity with impaired contractility. Data expression and other abbreviations are the same as in Table 1.

^a^By Kruskal-Wallis test or chi-square test.

In Table 1, we found that the incidence of diabetes mellitus was higher in the BOO group than in the bladder dysfunction group, and these may be associated with diabetes-related peripheral neuropathy of the bladder and impaired urethral relaxation during voiding^28^. Reduced activity of the nitric oxide pathway may contribute to the impaired urethral relaxation during voiding in diabetes patients^29^.

This study has limitations. The retrospective nature of this study may not represent the real prevalence of women with symptoms of voiding dysfunction in the general population. However, the large sample size study may make our data useful and more reliable. In addition, owing to the low ROC area of the Abrams-Griffiths number in predicting anatomic BOO, of the post-void residual in predicting bladder neck dysfunction and of the bladder contractility index in predicting bladder dysfunction, Abrams-Griffiths number, post-void residual and bladder contractility index may be not very helpful in determining cutoff values.

In conclusions, the videourodynamic findings of a large cohort of women with voiding dysfunction symptoms were presented, and can be used as initial guide for clinical management of female BOO.

## Methods

Between Oct 1997 and Jan 2015, medical records of women with complaints of voiding dysfunction (e.g. hesitancy, difficult urination, slow stream, intermittency, postmicturition leakage or urinary retention) who underwent videourodynamic studies at the Department of Urology of a medical center were reviewed. The symptoms of these patients were classified into 4 groups at the time-point of videourodynamic studies, including (1) voiding symptoms (including hesitancy, dysuria, slow stream or feeling of incomplete emptying), (2) urgency and/or urgency urinary incontinence, (3) frequency/nocturia, and (4) urinary retention (episodic or chronic). All patients had mild to severe voiding symptoms. However, only moderate and severe voiding symptoms were included in this retrospective analysis (Tables 1–3). The patients’ comorbidities, videourodynamic characteristics and urodynamic parameters were also recorded. Patients with a history of genitourinary tract cancer, overt neurogenic bladder dysfunction, high grade cystocele or prolapse, prior surgery for stress urinary incontinence, an established diagnosis of interstitial cystitis/painful bladder syndrome, chronic or active urinary tract infection were excluded. The diagnosis was made according to terminology from the International Continence Society^24, 30^. The institutional review board of the hospital approved this study. This study investigated the videourodynamic characteristics of female voiding dysfunction. No image that could lead to identification of a study participant will be published in the manuscript. The study was approved by the Institution Review Board of the hospital (IRB: 100-06). Informed consent was waived due to its’ retrospective analysis. The corresponding author confirmed that all methods were performed in accordance with relevant guidelines and regulations.

Videourodynamic studies were performed using multichannel urodynamic equipment (Life-Tech, Houston, TX, USA) and a C-arm fluoroscope (Toshiba, Tokyo, Japan) prior to any treatment. The procedure was performed in the sitting position with a 6 Fr dual-channel urethral catheter for recording the intravesical pressure, and the warm normal saline containing 20% urograffin was used for infusion. The intra-abdominal pressure was recorded using an 8 Fr rectal balloon catheter. The videourodynamic study was performed at a filling rate of 20–30 mL/min. The C-arm fluoroscope was positioned at 45 degrees from the buttocks so that the urethra could be lengthened and so that the bladder neck, urethral sphincter, and distal urethra could be clearly identified. Urethral sphincter electromyography was recorded using surface patch electromyography electrodes placed at the perianal area. The videourodynamic study was repeated at least once to demonstrate the reproducibility of the findings during the first examination. All descriptions and terminology for the urodynamic parameters were in accordance with the recommendations of the International Continence Society^24, 30^. Bladder capacity was derived from the sum of the voided volume and the post-void residual. Voiding efficiency was defined as the voided volume divided by bladder capacity.

A Pdet.Qmax of more than 35 cmH_2_O was considered a high Pdet.Qmax, while 10–35 cmH_2_O was considered a normal Pdet.Qmax, and 10 cmH_2_O or less was considered a low Pdet.Qmax^31^. Patients with a stable bladder, normal bladder sensation, a cystometric bladder capacity > 350 ml, a normal Pdet.Qmax or a low Pdet.Qmax but with a Qmax > 15 ml/s, and a post-void residual less than 10% of cystometric bladder capacity were considered urodynamically normal^31^. An acontractile detrusor is one that cannot be demonstrated to contract during urodynamic studies^30^. When patients had a Pdet.Qmax < 10 cmH_2_O and needed to void via abdominal straining or were unable to void, detrusor underactivity was diagnosed^32^. Detrusor hyperactivity and impaired contractility was defined as the presence of involuntary detrusor contraction during the filling phase and underactive detrusor function during the voiding phase, usually with a voiding efficiency of <50%^33, 34^.

Detrusor overactivity was defined as evidence of spontaneous detrusor contractions occurring during bladder filling or an uninhibited detrusor contraction occurring at cystometric capacity that usually resulting in voiding^24, 30^. If patients had a strong desire to void at a cystometric bladder capacity of less than 350 ml and without the occurrence of detrusor overactivity, they were considered to have bladder oversensitivity^24, 30^. However, some women diagnosed as detrusor underactivity or bladder oversensitivity had concomitant urgency and low amplitude detrusor overactivity (<5 cmH_2_O) during bladder filling, we did not classify these women to detrusor hyperactivity and impaired contractility or detrusor overactivity. Bladder compliance was measured as the incrementally increased cystometric volume at full bladder sensation divided by the increased detrusor pressure.

Women with voiding symptoms were classified as obstructed if there was radiographic evidence of obstruction between the bladder neck and distal urethra in the presence of a sustained detrusor contraction, which was usually associated with reduced or delayed urinary flow rate^5^. The final diagnosis of bladder neck dysfunction, dysfunctional voiding or poor relaxation of the external sphincter was made based on the main videourodynamic findings^4^. Voiding cystourethrography showed narrowing of the urethra at the proximal part in bladder neck dysfunction (Fig. 1B), the middle part in dysfunctional voiding (Fig. 1C), and at the distal part in poor relaxation of the external sphincter (Fig. 1D). In bladder neck dysfunction, besides a high voiding pressure and a low flow rate, a lack of significantly increased electromyography activity and a non-funneling appearance of the bladder neck on fluoroscopy were also noted on videourodynamics^3^. In dysfunctional voiding, intermittent electromyography activity was found during the voiding phase, causing an increase in voiding pressure^24^. In poor relaxation of the external sphincter, the electromyography activity did not decrease during attempts at voiding, resulting in a low voiding pressure or straining to void^24^. Cystoscopy was used in conjunction with the videourodynamic findings for differential diagnosis of the etiology of BOO^35^.

STATA software (Version 11.0; Stata Corp, College Station, TX, USA) was used for statistical analyses. The Kruskal-Wallis test and Wilcoxon rank-sum test were used as appropriate. A P value of less than 0.05 was considered statistically significant. The ROC curve analysis was performed to identify optimal cutoff values. The optimal cutoff value was determined by the point on the ROC curve that was closest to the upper left-hand corner.
